# The Book World of Medicine and Science

**Published:** 1901-08-10

**Authors:** 


### THE BOOK WORLD OF MEDICINE AND SCIENCE.

GUIDE TO SWITZERLAND. By W. A. B. COOLIDGE, formerly Editor of the *Alpine Journal*, with Cycling Supplement by Charles L. Freeston, with maps and illustrations. (London: Adam and Charles Black. 1901. Price 3s. 6d.)

WE have carefully compared this guide with our own experience and with other sources of information, and we have no hesitation in saying that it is admirably suited for its purpose, always supposing that this purpose is the instruction of the ordinary tourist who does not wish to go far off the beaten track. We doubt if anything will take the place of Bædeker—for those who want it—but there must be thousands of tourists every year for whom the minute directions therein given by which to reach all sorts of out-of-the way places are quite unnecessary, and yet who want something better than the little guide-books which are served out by the tourist agencies. The information given is throughout of a useful and practical character, and eminently suitable for those who wish to utilise the means of conveyance so lavishly provided for the tourist in most parts of this wonderful playground. It is to be noted that although the names of hotels are mentioned there is no attempt to indicate the prices charged or the comparative excellence of the various hostelries, the author getting rid of this thorny topic by referring his readers to an official publication, the *Hotels of Switzerland*, issued at Bale by the Association of Swiss Hotel Proprietors, the 1901 edition of which costs 20 cents within Switzerland or 40 cents within the Postal Union. The chapter on cycling is written in a very practical strain. The author points out a fact which will be interesting to the cyclist, namely, that it is chiefly where the railways have extended that the roads have tended to deteriorate, so that some of the very best of the roads from a cycling point of view are those which lead to places as yet not vulgarised by railways. What he says about brakes is worthy of consideration. While in England the brake is chiefly required for protection in sudden emergencies, in Switzerland one may have to keep it gently on for over twenty miles at a stretch, so that the sort of handle, as well as the sort of brake, becomes a matter of some importance. We have often been surprised to come across cyclists in the most unexpected quarters in Switzerland; the explanation being that road-making has there attained such a pitch of excellence and the gradients are so good, that the wheelman seems to get almost everywhere. This book is of very convenient size, is round cornered and soft in the pocket, and will, we think, be found a very useful companion.

SIDMOUTH AS A HEALTH RESORT AND PLACE OF RESIDENCE. By A. MACINDOE., M.D., D.P.H. (Bristol: John Wright and Co. 1901. Price 1s.)

THE author of this book commences with a plea for home, urging with some force the drawbacks which in many cases attend the removal of sick people to foreign watering places in search of health. He then goes on to describe the beauties of Sidmouth and to sing its praises, the latter not being a difficult task, for there is no doubt that to those whom Sidmouth suits the place is ideal. A good account is given of the geology and meteorology of the district, and of the water supply of the town, and a chapter is devoted to its medical aspect. Among the many diseases in which residence at Sidmouth is found to be beneficial we notice more particularly bronchitis, kidney diseases, and heart disease. For the treatment of the latter by the method originated at Nauheim, special arrangements have been made. For convalescents from various tropical diseases, and for elderly people who wish for a safe climate, Sidmouth seems peculiarly suitable.

SOME ADVERTISING GUIDE BOOKS.

AMONG the various guide books which cover our table, a not inconsiderable number may, we think, be properly regarded as advertisements; that is, they evidently have for their motive the attraction of people to certain places or to certain routes rather than the mere giving of information to those who want it. It is only right to say, however, that as guide books they are none the worse for that. First among books of this class we would place the admirably illustrated handbooks issued by the Great Eastern Railway. "Holidays in Eastern Counties," edited by Percy Lindley, is a very well mounted description of what is to be seen in the districts served by this railway. Nicely written and charmingly illustrated, we have no doubt it will lead many people to spend their holidays among the scenes which it describes. The "Tourist's Guide to the Continent," issued by the same company, gives a large amount of information and a good deal of pleasantly-written gossip about the various places which may be reached by the fine steamers which start from Harwich every evening—practically all Europe—while "Rhineland," also edited by Percy Lindley, deals more particularly with the Rhine country.

The London and South-Western Railway Company also issues a series of illustrated local guides. The district which this railway serves contains scenes of beauty unsurpassed in England and these penny guides will no doubt serve to remind people of what there is to be seen down the line.

"Autumn Holidays" is an admirably illustrated pamphlet full of tempting suggestions for those embarking on holiday travel. It is issued by Messrs. Lunn and Perowne, and is intended to show how to get to everywhere by aid of their touring system, which it does with great success.

For cyclists in England the "Hovis Cycle Road Map," issued in sections, which is intended not merely to show the way but to advertise Hovis Bread and Ariel cycles, will be found acceptable. It is clearly printed, markings are introduced to indicate the varying gradients of some of the hills, and much useful information is given in regard to matters interesting to cyclicts.

A hand-book very full of information about tours to Norway and the Orkney and Shetland Isles has been issued by the North of Scotland and Orkney and Shetland Steam Navigation Company by which the well-known St. Sunniva is run.

"Weymouth: a Guide and Souvenir," issued by the Town Council of that rising watering-place, gives a good idea of what may be seen not only in the town, but in the district round about. The little book is full of illustrations, and may be obtained post free on application to the town clerk. The same may be said of a similar guide to Deal, also officially published. A considerable number of other guide books of this class might be mentioned if space allowed; in fact the time would seem to be approaching when we shall look as a matter of course to receiving along with our railway ticket a full supply of guide books, maps, and all conceivable kinds of information about any place we go to for our holidays.

